# Stimulation in primary and secondary metabolism by elevated carbon dioxide alters green tea quality in *Camellia sinensis* L

**DOI:** 10.1038/s41598-017-08465-1

**Published:** 2017-08-11

**Authors:** Xin Li, Lan Zhang, Golam Jalal Ahammed, Zhi-Xin Li, Ji-Peng Wei, Chen Shen, Peng Yan, Li-Ping Zhang, Wen-Yan Han

**Affiliations:** 1 Key Laboratory of Tea Biology and Resources Utilization, Ministry of Agriculture, Tea Research Institute, Chinese Academy of Agricultural Sciences, 9 Meiling Road, Hangzhou, 310008 P.R. China; 2 Department of Horticulture, Zijingang Campus, Zhejiang University, Yuhangtang Road 866, Hangzhou, 310058 P.R. China; 3 Graduate School of Chinese Academy of Agricultural Sciences, Beijing, P. R. China

## Abstract

Rising CO_2_ concentration, a driving force of climate change, is impacting global food security by affecting plant physiology. Nevertheless, the effects of elevated CO_2_ on primary and secondary metabolism in tea plants (*Camellia sinensis* L.) still remain largely unknown. Here we showed that exposure of tea plants to elevated CO_2_ (800 µmol mol^−1^ for 24 d) remarkably improved both photosynthesis and respiration in tea leaves. Furthermore, elevated CO_2_ increased the concentrations of soluble sugar, starch and total carbon, but decreased the total nitrogen concentration, resulting in an increased carbon to nitrogen ratio in tea leaves. Among the tea quality parameters, tea polyphenol, free amino acid and theanine concentrations increased, while the caffeine concentration decreased after CO_2_ enrichment. The concentrations of individual catechins were altered differentially resulting in an increased total catechins concentration under elevated CO_2_ condition. Real-time qPCR analysis revealed that the expression levels of catechins and theanine biosynthetic genes were up-regulated, while that of caffeine synthetic genes were down-regulated in tea leaves when grown under elevated CO_2_ condition. These results unveiled profound effects of CO_2_ enrichment on photosynthesis and respiration in tea plants, which eventually modulated the biosynthesis of key secondary metabolites towards production of a quality green tea.

## Introduction

Climate change is one of the most important complex factors that greatly impacts global food production. It is predicted that effect of climate change will be intensified over time. For instance, the concentration of atmospheric CO_2_, an important parameter of climate change, has been increased tremendously in the last century and will be doubled at the end of 21^st^ century (IPCC 2007)^1^. Studies have revealed that rising atmospheric CO_2_ concentrations greatly influence plant growth and responses to biotic and abiotic stresses^2–4^. The general interpretation in favour of rising CO_2_ is that elevated CO_2_ stimulates photosynthesis in plants that eventually results in increased yield in terms of quantity. Recent studies have also revealed that plants grown under elevated CO_2_ maintain a consistently higher leaf dark respiration (mitochondrial respiration), compared with that of ambient CO_2_^5^. Elevated CO_2_-simulated enhanced respiration can increase crop yield, by providing greater energy to export photoassimilate from source leaves to sink tissues^5, 6^.

Photosynthesis plays an important role in plant metabolism by synthesizing photoassimilates that are used as substrates for all other biosynthetic pathways^7^. Respiration utilizes photoassimilate as substrate to generate C-skeleton intermediates, reductants such as NADH and NADPH, and usable energy i.e. ATP as products^8^. The energy provided by respiration is the source energy for secondary metabolism and the products of respiration serve as the synthetic precursors of secondary metabolites^9^. Two main biochemical processes such as ribulose-1,5-bis-phosphate (RuBP) carboxylase/oxygenase (RuBisCO) carboxylation and RuBP regeneration strictly control the rate of photosynthesis^6^. Elevated CO_2_ not only increases activity of RuBisCO to enhance photosynthetic rate, but also alters partitioning of the photoassimilates for the biosynthesis of secondary metabolites^10^. Moreover, elevated CO_2_ increases concentration of non-structural carbohydrate that may stimulate secondary metabolism in plants^10, 11^. Nonetheless, mitochondrial respiration plays a key role in optimizing adequate photosynthetic rates in plants^6^. Prior studies showed that increased carbohydrate availability and energy demand under elevated CO_2_ enhance respiration rate, which helps plant to optimize the allocation of carbon and nutrient for maximizing photosynthesis and plant growth^5^.

Tea is a fascinating health drink, extensively consumed for its health benefits and astringenic property around the world. Green tea is typically produced from two leaves and a bud of perennial tree tea [*Camellia sinensis* (L.) O. Kuntze]. The health benefits of green tea and its pleasant taste are due to presence of bioactive compounds predominantly derived from secondary metabolic pathway^12–14^. The composition of primary metabolite and secondary metabolites determines the ultimate quality of green tea^14^. Although a number of previous studies have showed that elevated CO_2_ influences both primary and secondary metabolism in a range of plant species^10, 15, 16^, one crucial topic that has been ignored is the effect of elevated CO_2_ on the growth of tea plants and production of secondary metabolites involved in tea quality.

Mostly two groups of chemicals such as tea polyphenols (TP) and amino acids (AA) are considered as main determinants of the taste or pleasant flavor of tea. Catechins are major TP that significantly influence the flavor of green tea, while theanine, an abundant non-protein AA in tea leaves is responsible for its umami taste^17^. Catechins are well known for its role in preventing cancer, cardiovascular, neurodegenerative and other oxidative stress-related diseases^18^. Given that catechins are flavan-3-ol type of flavonoid, its synthesis involves participation of the phenylpropanoid and flavonoid pathways. Theanine is used as one of the biosynthetic precursors of catechins^19^. Health benefits of theanine include reduction of high blood pressure, induction of relaxation and inhibition of the side effects of caffeine^17, 19^. Caffeine, a secondary metabolite belongs to purine alkaloids, is synthesized in tea plants from purine nucleotides^12^. The concentration of caffeine in plants is high in young leaves and flowers compared with other plant parts^12, 20, 21^. Although a moderate amount of caffeine has stimulatory effects on human health, its excessive consumption is often associated with health hazards such as sleep deprivation, tachycardia, abortion and miscarriages^22^. Therefore, caffeine level in a quality tea is expected to be minimum, so that its consumption would not exceed total dietary threshold. It is evident that biosynthesis of these secondary metabolites that are the key determinants of tea quality occurs through complex as well as inter-connected metabolic pathways that often converge between primary and secondary metabolism. However, the effects of elevated CO_2_ on the concentrations of tea secondary metabolites and expression of their regulatory genes still remain elusive.

Unlike annual crops, tea plants remain in active production for a long period of time, even for hundred years, which may allow them to experience climate change over the century^23^. It is believed that long life span of tea plants may lead them to operate massive physiological adaptation instead of genetic modification. Therefore, in the current study, we intend to investigate potential changes in some primary metabolic processes such as photosynthesis and respiration following exposure of tea plants to elevated CO_2_ for a period of 24 days. In addition, we analyzed the concentrations of various primary metabolites and tea quality-related secondary metabolites coupled with the expression of key genes involved in their biosynthetic pathways. It was hypothesized that elevated CO_2_ would alter the yield and quality of tea by modulating the primary and secondary metabolism in tea leaves. The results of this study will help us to better understand the preliminary response of tea plants to elevated CO_2_ at physiological and molecular levels.

## Results

### Exposure of tea seedlings to elevated CO_2_ enhances plant growth and biomass accumulation

Many experimental studies have shown that elevated CO_2_ conditions stimulate plant growth and biomass production in a wide range of plant species^2, 11, 15, 16^. To clarify this assumption in tea, we exposed tea seedlings to ambient CO_2_ and elevated CO_2_ conditions for 24 days. Results showed that elevated CO_2_ not only increased plant height (by 13.46%), but also promoted dry weights of shoot and root by 24.68 and 67.80%, respectively (Table 1). A positive stimulation in both shoot and root biomass accumulation by elevated CO_2_ eventually resulted in an increased root to shoot ratio by 27.66% compared with that in ambient CO_2_.

**Table 1:** Effect of elevated CO_2_ concentration (800 µmol mol^−1^ for 24 d) on growth and biomass production in tea seedlings.

Treatments	Plant height (cm)	Leaf DW seedling^−1^ (g)	Stem DW seedling^−1^ (g)	Shoot DW seedling^−1^ (g)	Root DW seedling^−1^ (g)	Ratio of Root to Shoot
Ambient CO_2_	55.7 ± 3.73 b	4.8 ± 1.26 b	7.7 ± 0.98 b	12.5 ± 2.24 b	5.9 ± 0.74 b	0.47 ± 0.031 b
Elevated CO_2_	63.2 ± 4.65 a	7.0 ± 1.33 a	9.6 ± 1.47 a	16.7 ± 2.8 a	9.9 ± 1.32 a	0.60 ± 0.064 a

Mean denoted by different letters indicate significant differences between the treatments (*P* < *0*.*05*). DW, Dry weight.

### Elevated CO_2_ promotes photosynthesis by increasing RuBisCO carboxylation and regeneration capacity

**Figure 1:**
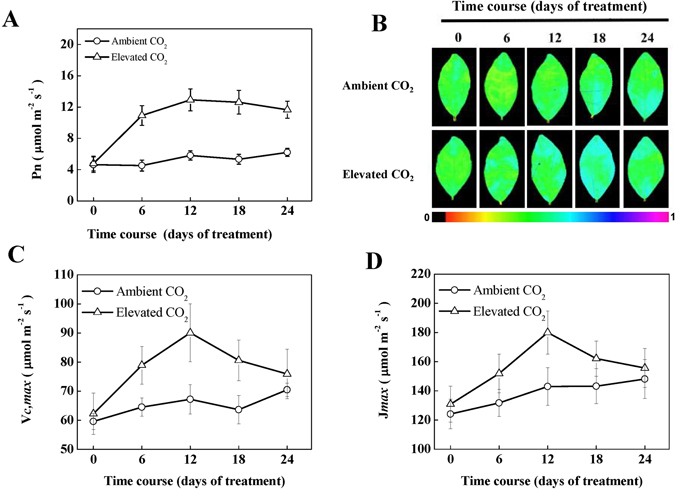
Photosynthetic response of tea plants to elevated CO_2_. (**A**) Net photosynthetic rate (Pn), (**B**) the quantum efficiency of photosystem II (Φ_PSII_), false colour code depicted in the image ranges from 0 (black) to 1(purple), (**C**) maximum carboxylation rate of RuBisCO (*V*_*cmax*_), (**D**) maximum rates of RuBP regeneration (*J*_*max*_). Tea seedlings were exposed to either ambient (380 µmol mol^−1^) or elevated CO_2_ concentration (800 µmol mol^−1^) for 24 days. Measurements were taken at different time-points as mentioned in the respective figures. The results are expressed as the mean values ± SD, n = 6.

To examine whether increased biomass accumulation under elevated CO_2_ is associated with photosynthetic performance of tea, we measured net photosynthetic rate at 5 time points over 24 days. Results showed that exposure of plants to elevated CO_2_ rapidly increased Pn that eventually reached maximum level at 12 day, and then remained more or less stable up to 24 day, indicating an acclimation response of CO_2_ assimilation capacity to elevated CO_2_ after 12 day exposure (Fig. 1A). Specifically, elevated CO_2_ increased Pn by 141.98, 122.25, 136.93 and 87.90% at 6, 12, 18 and 24 day, respectively as compared with that in ambient CO_2_. We also analyzed the quantum efficiency of PSII photochemistry (Φ_PSII_) that represents photosynthetic efficiency of tea leaves. Pseudo color images of Φ_PSII_ were shown in Fig. 1B. It is noticeable that the Φ_PSII_ remained stable over the experimental period in tea plants grown under ambient CO_2_ concentration. However, in tea plants that were grown under elevated CO_2_, Φ_PSII_ tended to increase, reaching the highest value at 18 d following exposure to elevated CO_2_. Afterward, Φ_PSII_ declined slightly. In addition, *Vcmax* increased until 12 day when it reached the highest peak, and then tended to decline over time (Fig. 1C). Similar trend was observed for *Jmax* under elevated CO_2_ condition, although notable difference was only found at 12 day under elevated CO_2_ treatment as compared with that in ambient CO_2_ (Fig. 1D).

### Elevated CO_2_ increases respiration by increasing O_2_ uptake

**Figure 2:**
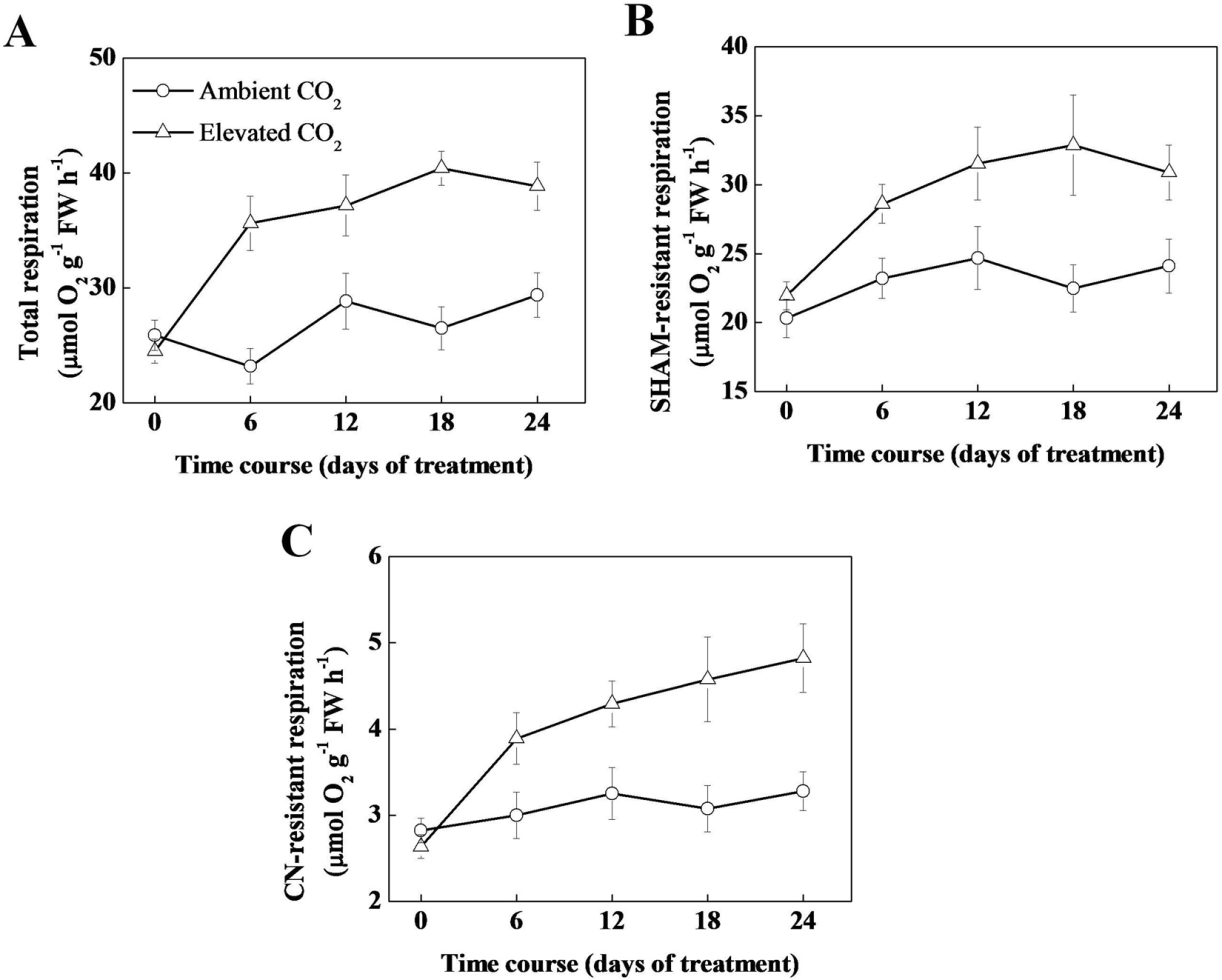
Changes in the O_2_ uptake rate of tea seedlings grown at ambient (380 µmol mol^−1^) or elevated CO_2_ concentration (800 µmol mol^−1^). (**A**) Total respiration, (**B**) salicylhydroxamic acid (SHAM)-resistant respiration, (**C**) cyanide (CN)-resistant respiration. Tea seedlings were exposed to either ambient (380 µmol mol^−1^) or elevated CO_2_ concentration (800 µmol mol^−1^) for 24 day. Measurements were taken at different time-points as mentioned in the respective figures. The results are expressed as the mean values ± SD, n = 6.

An optimum balance between photosynthesis and respiration is required for proper biomass accumulation^6, 8^. To check whether elevated CO_2_ also affects respiration, we measured total respiration, SHAM-resistant respiration and CN-resistant respiration. Compared with ambient CO_2_, elevated CO_2_ increased total respiration rate by 53.63, 28.88, 52.67 and 32.29% at 6, 12, 18 and 24 day, respectively (Fig. 2A). Similarly, elevated CO_2_ increased SHAM-resistant respiration by 23.22, 27.70, 46.22 and 28.13% and CN-resistant respiration by 29.61, 32.04, 48.83 and 47.11%, respectively (Fig. 2B). Unlike Pn, maximum total respiration was recorded at 18 d, although respiration rates recorded at 12, 18 and 24 day were not much different, indicating a respiratory acclimation response to elevated CO_2_. While SHAM-resistant respiration remained stable after 12 day, CN-resistant respiration showed an increasing trend even at 24 day under elevated CO_2_ (Fig. 2C). Taken together, from the beginning to the end of the experiment, the rates of total respiration, SHAM-resistant and CN-resistant respiration were higher in tea plants grown under elevated CO_2_ than that under ambient CO_2_.

### Effect of elevated CO_2_ on concentration of sugar, starch, carbon and nitrogen

**Figure 3:**
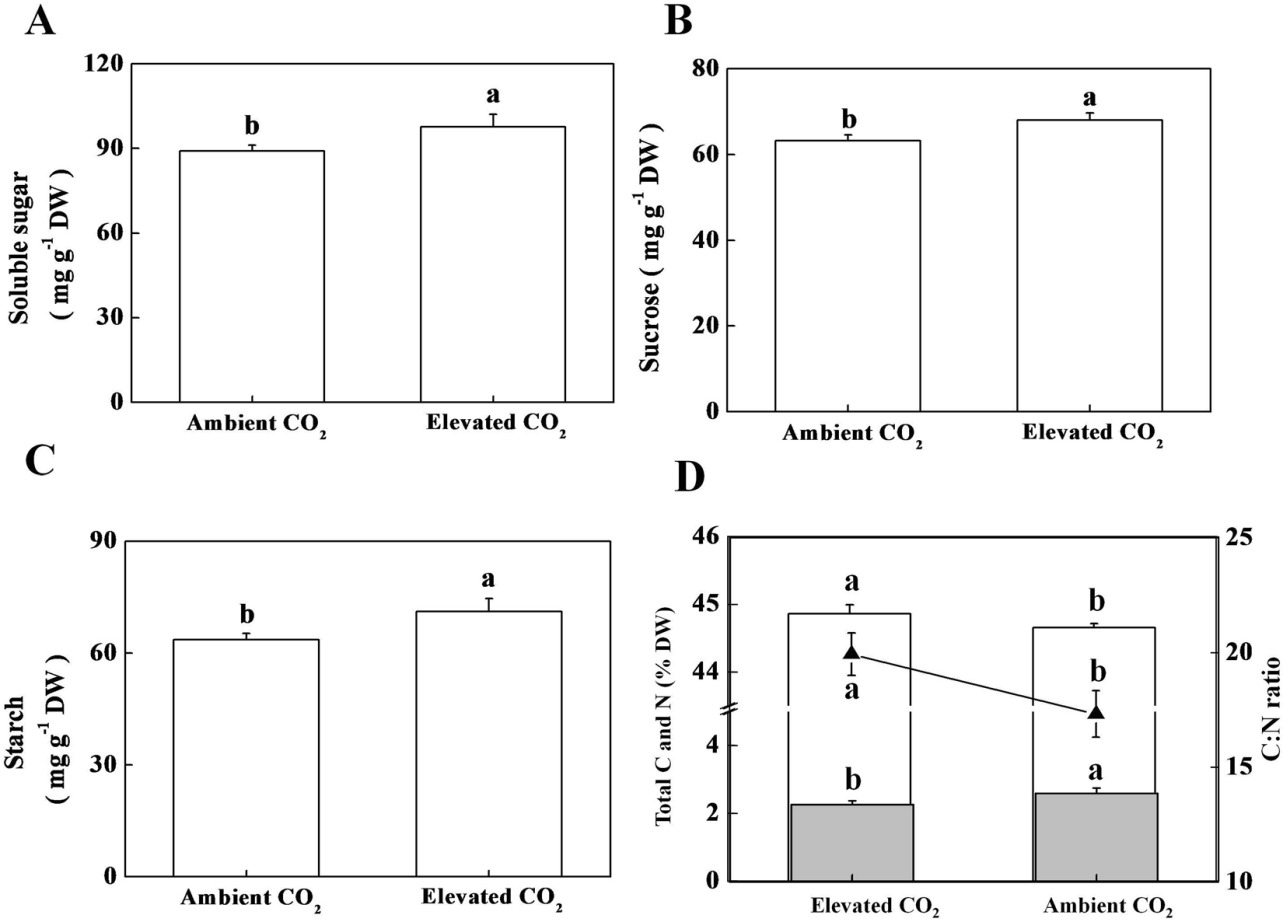
Effect of ambient (380 µmol mol^−1^) or elevated CO_2_ concentration (800 µmol mol^−1^) on carbohydrate, carbon and nitrogen concentration in tea leaves. (**A**) Soluble sugar concentration, (**B**) Sucrose concentration, (**C**) Starch concentration, and (**D**) Total carbon, total nitrogen and C:N ratio. Histograms with solid fill color (■) and without fill color (□) represent total nitrogen and total carbon, respectively, while line graph (-▲-) represents C: N ratio. Leaf samples were harvested after exposure of tea plants to different concentrations of atmospheric CO_2_ for 24 days. Data are the means of four replicates (±SD). Mean denoted by different letters indicate significant differences between the treatments (*P* < *0*.*05*).

As elevated CO_2_ stimulated both photosynthesis and respiration in tea leaves, we then looked into carbon and nitrogen metabolism in tea leaves. Elevated CO_2_ significantly increased the concentration of sugar, sucrose and starch (Fig. 3A–C). While concentration of total carbon increased in tea leaves under elevated CO_2_, concentration of total nitrogen decreased (Fig. 3D). Such changes in total C and total N eventually resulted in an increased C: N ratio in tea leaves under elevated CO_2_ conditions.

### Effect of CO_2_ enrichment on tea quality attributes

**Figure 4:**
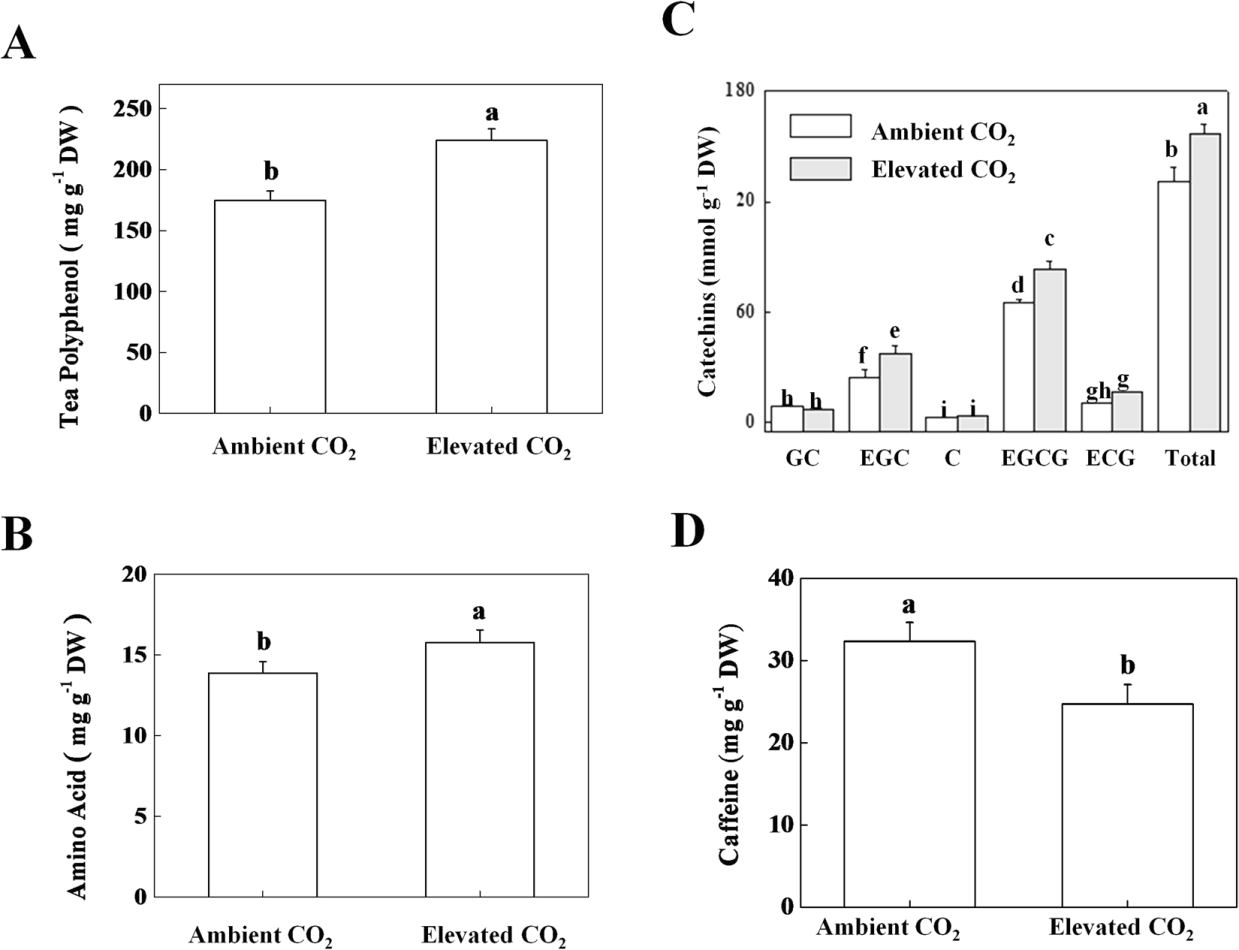
Changes in polyphenol, amino acid, and caffeine concentration in leaves of tea seedlings grown at ambient (380 µmol mol^−1^) or elevated CO_2_ concentration (800 µmol mol^−1^). (**A**) Total tea polyphenol, (**B**) total amino acids, (**C**) Individual catechins, and (**D**) Caffeine concentrations. Leaf samples were harvested after exposure of tea plants to different concentrations of atmospheric CO_2_ for 24 days. Data are the means of four replicates (±SD). Mean denoted by different letters indicate significant differences between the treatments (*P* < *0*.*05*).

Impact of elevated CO_2_ on tea quality attributes is largely unknown. We determined key bioactive compounds in tea leaves that are responsible for tea quality. Under elevated CO_2_, total tea polyphenol and amino acid concentration increased by 28.21 and 13.49%, respectively (Fig. 4A and B), while caffeine concentration decreased by 23.64% as compared with that under ambient CO_2_ (Fig. 4D). We also quantified individual catechins and amino acids concentrations in tea leaves. Results showed that (-)-gallocatechin (GC) and (-)-catechin (C) concentrations were not altered by CO_2_ enrichment; however, (-)-epigallocatechin (EGC) and (-)-epigallocatechin-3-gallate (EGCG) concentrations were significantly increased following CO_2_ enrichment, resulting in an overall increase in total catechins content under elevated CO_2_ (Fig. 4C). Likewise, individual amino acid concentration was differentially modulated by elevated CO_2_ in tea leaves (Table 2). The concentrations of aspartic acid, theanine, proline, alanine and phenylalanine increased, while that of threonine and serine decreased following exposure of tea plants to elevated CO_2_. Meanwhile, the concentrations of glutamic acid, glycine, valine, isoleucine, tyrosine, histidine, lysine and arginine were not affected by CO_2_ enrichment treatment (Table 2).

**Table 2:** Effect of elevated CO_2_ concentration (800 µmol mol^−1^ for 24 d) on amino acids concentration in tea leaves.

Amino acids (mg g^−1^ DW)	Ambient CO_2_	Elevated CO_2_
Aspartic acid (Asp)	1.124 ± 0.013 b	1.730 ± 0.029 a
Threonine(Thr)	0.672 ± 0.005 a	0.448 ± 0.007 b
Serine(Ser)	0.593 ± 0.006 a	0.467 ± 0.007 b
Glutamic acid(Glu)	1.394 ± 0.021 a	1.812 ± 0.017 a
L-Theanine (Thea)	14.336 ± 0.571 b	22.624 ± 0.685 a
Proline (Pro)	0.535 ± 0.011 b	0.827 ± 0.009 a
Glycine(Gly)	0.224 ± 0.008 a	0.218 ± 0.005 a
Alanine(Ala)	0.448 ± 0.015 b	0.677 ± 0.022 a
Valine (Val)	0.856 ± 0.014 a	0.861 ± 0.009 a
Isoleucine (Ile)	0.433 ± 0.008 a	0.427 ± 0.014 a
Tyrosine (Tyr)	0.841 ± 0.031 a	0.863 ± 0.024 a
Phenylalanine(Phe)	0.540 ± 0.011 b	0.618 ± 0.019 a
Histidine (His)	0.224 ± 0.004 a	0.228 ± 0.006 a
Lysine (Lys)	0.448 ± 0.010 a	0.453 ± 0.014 a
Arginine (Arg)	0.672 ± 0.013 a	0.683 ± 0.009 a

Mean denoted by different letters indicate significant differences between the treatments (*P* < *0*.*05*). DW, Dry weight.

### Changes in the expressions of catechin, caffeine and theanine synthesis genes under elevated CO_2_

**Figure 5:**
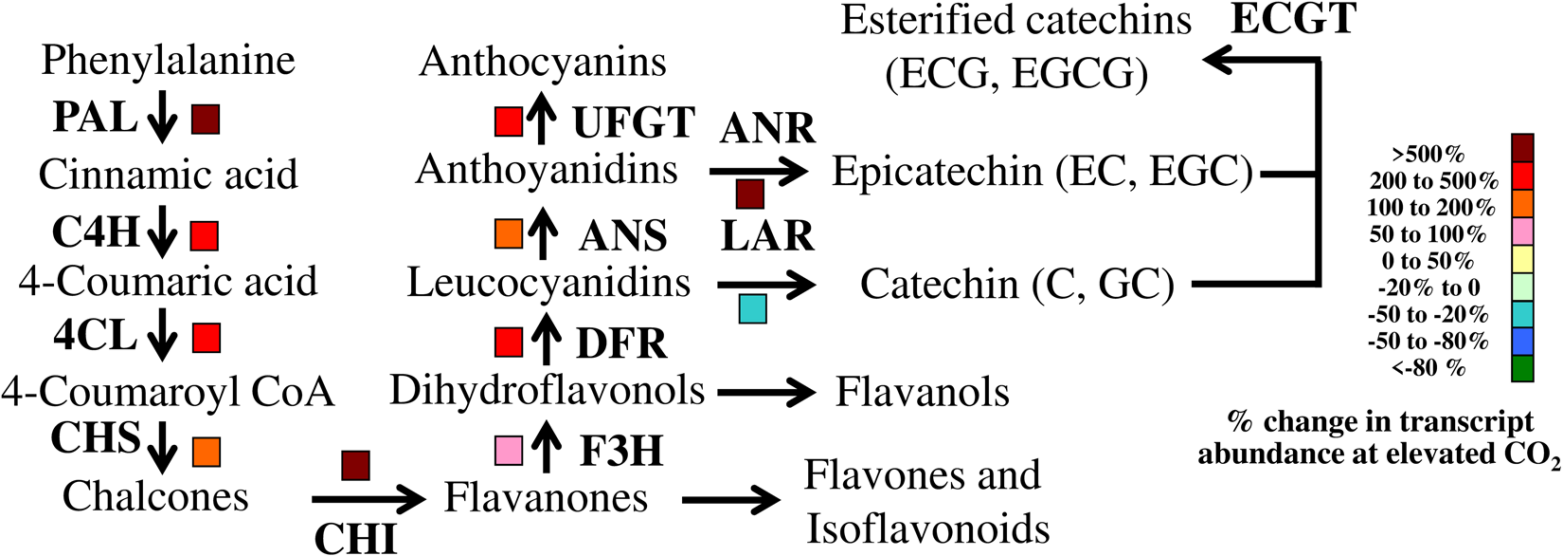
Transcript levels of catechins synthetic pathway-related genes in tea leaves as influenced by ambient (380 µmol mol^−1^) or elevated CO_2_ concentration (800 µmol mol^−1^). Leaf samples were harvested at 24 days following exposure of tea seedlings to different atmospheric CO_2_ concentrations. Expression levels of genes were analyzed by qPCR using gene-specific primer pairs (Supplementary Table S1). Four biological replicates were used for qPCR analysis.

As we found an increased catechins concentration under elevated CO_2_, we anticipated that increased concentration of catechins might be attributed to increased biosynthesis of catechins. Therefore, we analyzed expression of key genes in catechins synthesis pathway, such as *PHENYLALANINE AMMONIA*-*LYASE* (*CsPAL*), *CINNAMATE 4*-*HYDROXYLASE* (*CsC4H*), *P*-*COUMARATE*:*COA LIGASE* (*Cs4CL*), *CHALCONE SYNTHASE* (*CsCHS*), *CHALCONE ISOMERASE* (*CsCHI*), *FLAVANONE 3*-*HYDROXYLASE* (*CsF3H*), *DIHYDROFLAVONOL 4*-*REDUCTASE* (*CsDFR*), *ANTHOCYANIDIN SYNTHASE* (*CsANS*), *UDP*- *GLUCOSE FLAVONOID 3*-*O*-*GLUCOSYL TRANSFERASE* (*CsUFGT*), *ANTHOCYANIDIN REDUCTASE* (*CsANR*) and *LEUACOANTHOCYANIDIN REDUCTASE* (*CsLAR*) by real-time quantitative polymerase chain reaction (qPCR). As shown in Fig. 5, elevated CO_2_ treatment caused an induction in the gene expression in all steps of the catechins biosynthetic pathway except for *CsLAR*. For instance, gene expression levels of *CsPAL* and *CsANR*, the first and last regulatory genes, respectively, in catechins biosynthetic pathway were upregulated by 5 fold under elevated CO_2_ as compared with that under ambient CO_2_. In contrast, transcript of *CsLAR* was down-regulated by 50% under elevated CO_2_. Transcript data are more or less in accordance with the endogenous content of individual catechins, implying that elevated CO_2_ influences catechins biosynthesis at transcription level.

**Figure 6:**
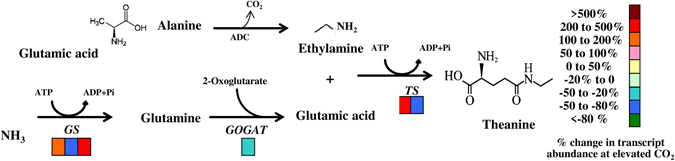
Expression of theanine synthetic pathway-related genes in tea leaves as influenced by ambient (380 µmol mol^−1^) or elevated CO_2_ concentration (800 µmol mol^−1^). Leaf samples were harvested at 24 days following exposure of tea seedlings to different atmospheric CO_2_ concentrations. Expression levels of genes were analyzed by qPCR using gene-specific primer pairs (Supplementary Table S1). Four biological replicates were used for qPCR analysis.

Theanine is the major tea amino acids accounting for more than 50% of total free amino acid in tea^13^. To assess whether increased amino acid content under elevated CO_2_ was attributed to theanine biosynthesis, we analyzed the key genes of theanine synthesis pathway such as *GLUTAMINE SYNTHETASE* (*CsGS*), *GLUTAMINE*: *2*-*OXOGLUTARATE AMINOTRANSFERASE* (*CsGOGAT*) and *THEANINE SYNTHASE* (*CsTS*). Except for *CsGOGAT*, expression levels of *CsGS* and *CsTS* were upregulated under elevated CO_2_, indicating that CO_2_ enrichment induced transcription of theanine biosynthetic genes that not only increased content of theanine, but also promoted total free amino acid content in tea leaves (Fig. 6).

**Figure 7:**
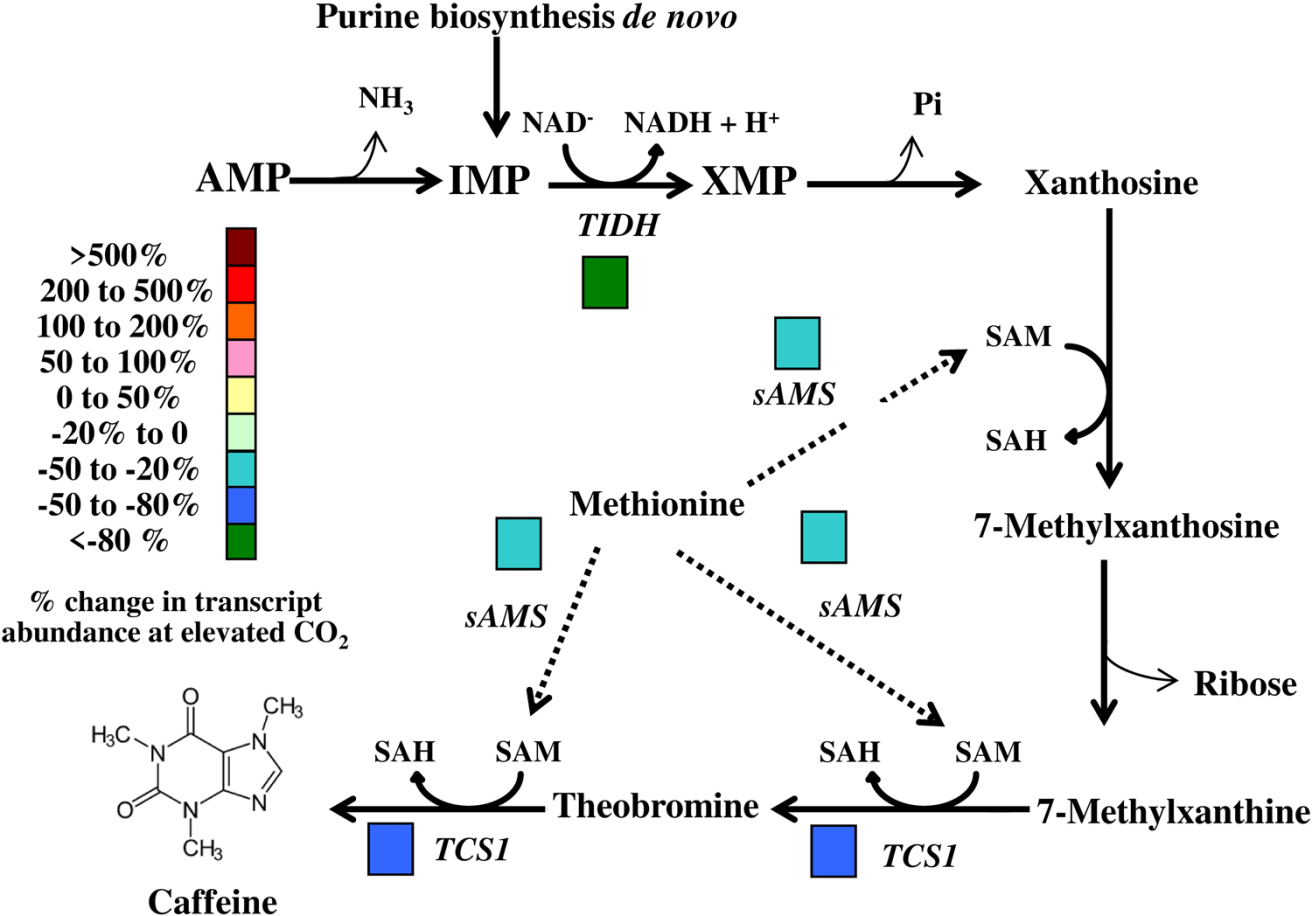
Transcriptional response of caffeine biosynthetic genes to elevated CO_2_ in tea leaves. Tea seedlings were exposed to either ambient (380 µmol mol^−1^) or elevated CO_2_ concentration (800 µmol mol^−1^) for 24 days. Expression levels of genes were analyzed by qPCR using gene-specific primer pairs (Supplementary Table S1). Four biological replicates were used for qPCR analysis.

Finally, we analyzed transcript levels of caffeine synthesis genes such as *INOSINE 5’*-*MONOPHOSPHATE DEHYDROGENASE* (*TIDH*), *S*-*ADENOSYL*-*L*-*METHIONINE SYNTHASE* (*sAMS*) and *TEA CAFFEINE SYNTHASE 1* (*TCS1*) following exposure of tea seedling to elevated CO_2_ for 24 d. Unlike catechins and theanine, genes relating to caffeine synthesis were down-regulated under elevated CO_2_ (Fig. 7). For instance, transcription of *TIDH*, the gene involved in encoding TIDH that catalyzes degradation of adenine nucleotides (AMP route) to xanthosine AMP (XAMP route), was decreased by approx. 80% under elevated CO_2_. Likewise, expression of *sAMS* gene, which is typically involved in supplying *S*-adenosl-L-methionine (SAM) from methionine, was also down-regulated by 20–50% under elevated CO_2_ condition. Consistently, expression of *TCS1* that encodes caffeine synthase, the enzyme that catalyzes final two conversions steps of caffeine biosynthesis, was down-regulated by 80% under elevated CO_2_. Down-regulated expression of caffeine biosynthetic genes under elevated CO_2_ was in full agreement with the decreased concentration of caffeine in tea leaves.

## Discussion

Rising atmospheric CO_2_ concentrations have a profound effect on plant growth, development and responses to stresses^2, 3, 15, 16^. While impact of elevated CO_2_ has been extensively studied in major food crops, its effect on yield and quality of important beverage crops such as tea remained largely unknown^23^. In this study, we exposed tea seedlings to elevated level of CO_2_ for a period of 24 days and monitored primary metabolism-related processes such as photosynthesis and respiration at different time-points. Results showed that CO_2_ enrichment improved both photosynthesis and respiration in tea plants, albeit a photosynthetic acclimation response was noticed after 6 day exposure. On one hand, elevated CO_2_ increased photosynthesis and respiration towards increased biomass accumulation, while one the other hand, enhancement in photosynthesis and respiration perhaps altered resource allocation towards secondary metabolism, leading to an increased biosynthesis of tea total polyphenols (TP), amino acids (AA), catechins and theanine, but a decreased content of caffeine. qPCR analysis of the catechins, theanine and caffeine biosynthetic genes further confirmed the stimulatory effects of elevated CO_2_ at transcriptional level. Our results suggest that rising CO_2_, a driving force of climate change not only improves primary metabolism, but also promotes secondary metabolism towards production of a quality green tea.

In Arabidopsis, elevated CO_2_ causes a metabolic perturbation that compels plants to increase its functions or activity by consuming or storing photoassimilates^16^. In the current study, elevated CO_2_ might also increase production and consumption of photoassimilates in tea plants by enhancing net photosynthesis and respiration rate, respectively (Figs 1–3). It is to be noted that an enhancement in photosynthesis under elevated CO_2_ could provide increased levels of substrates for glycolysis and a significant increase in TCA cycle intermediates might contribute to increased C-partitioning to respiration or for other relevant anabolic pathways^16^. However, a photosynthetic acclimation response was noticed following 12 day CO_2_ enrichment (Fig. 1). Earlier studies showed that exposure of plants to long-term CO_2_ enrichment may induce photosynthetic acclimation^24^, which is in agreement with our current observation. The acclimation response of Pn, was more or less accompanied with values of Φ_PSII_, *Vcmax* and *Jmax*. As Pn is dependent on RuBisCO carboxylation and RuBP regeneration rate^6, 15^, a close association between Pn, *Vcmax* and *Jmax* suggests that elevated CO_2_ perhaps stimulates RuBisCO carboxylation and RuBP regeneration rate to positively affect CO_2_ assimilation rate. Importantly, elevated CO_2_ increased plant growth in tea plants (Table 1). An increased plant growth due to elevated CO_2_ may stimulate growth respiration proportionally^6^. In addition, an enhancement in photosynthesis by elevated CO_2_ may increase carbohydrate availability and energy demand which necessitate plant to increase its respiration rate^5^. Therefore, the enhanced respiration rate under elevated CO_2_ was attributed to increased photosynthetic rate in tea plants (Fig. 1).

In the current study, CO_2_ enrichment remarkably increased contents of polyphenols including catechins (Fig. 4). The biosynthesis of catechins through phenylpropanoid and flavonoid pathways is dependent on the primary metabolism that supplies initial compounds required to run phenylpropanoid pathway^14, 25^. We found that elevated CO_2_ increased primary metabolites such as sugar, sucrose and starch in tea leaves (Fig. 3A–C). Moreover, carbon to nitrogen ratio was increased in tea leaves under elevated CO_2_ (Fig. 3D). As per carbon-nutrient balance theory, CO_2_ enrichment increases the carbon to nitrogen ratio and thus a greater amount of carbohydrates can be allocated to secondary metabolism in plants^26^. In addition, many experimental studies have shown that elevated CO_2_ conditions increase carbon-rich structural compounds and secondary metabolites in a range of plant species^1, 10, 15, 16^. It is worth mentioning that catechins are C-rich secondary metabolites. As C capture through photosynthesis was remarkably induced under elevated CO_2_, it is highly likely that increased C supply towards secondary metabolic pathway can be a potential reason for increased production of C-based secondary metabolites such as catechins under elevated CO_2_ condition.

To get a better insight into elevated CO_2_-modulated catechins biosynthesis, we analyzed the transcript levels of key genes of catechins biosynthetic pathway. The first committed step in the biosynthesis of catechins, is deamination of L-phenylalanine to *trans* cinnamic acid, catalyzed by the enzyme PAL. PAL is encoded by *CsPAL* in tea^25^. In the current study, consistent with catechins content, gene expression level of *CsPAL* was upregulted by 5-fold under elevated CO_2_ condition (Fig. 5). In tobacco, elevated CO_2_ (1000 ppm) significantly increased activity of PAL at both lower- and higher N-supply^10^. However, the effect of elevated CO_2_ on PAL activity was more pronounced at the lower N-supply. In case of tea, N-deficiency leads to increased accumulation of catechins especially epicatechins, which was associated with upregulated expression of *CsPAL* and other key genes (*CsCHS*, *CsCHI*, *CsDFR*, *CsANS* and *CsANR*) in catechin biosynthetic pathway^27^. In Arabidopsis, effect of short-term elevated CO_2_ on expression of genes involved in nitrogen metabolism may resemble the perturbation caused by N-deficiency^16^. In the current study, total nitrogen concentration in tea leaves was decreased under elevated CO_2_ (Fig. 3D). Therefore, it is quite plausible that elevated CO_2_-induced enhanced photosynthesis and/or perturbed N-metabolism might lead to increased production of catechins in tea plants.

Notably, except for *CsLAR*, other key regulatory genes in catechins biosynthetic pathway such as *CsC4H*, *Cs4CL*, *CsCHS*, *CsCHI*, *CsF3H*, *CsDFR*, *CsANS*, *CsUFGT* and *CsANR* all were upregulated under elevated CO_2_ (Fig. 5). At the final step of catechins biosynthesis, *CsLAR* catalyzes conversion of leucocyanidins into catechins (C, GC), while *CsANR* catalyzes conversion of anthoyanidins into epicatechins (EC, EGC)^25^. In line with suppression of *CsLAR* expression, the concentrations of GC and C were slightly decreased or remained unaltered, respectively under elevated CO_2_ in tea leaves (Fig. 4C). By contrast, upregulation of *CsANR* under elevated CO_2_ resulted in increased EGC concentration. Subsequently, gallylation of epicatechins caused an increased accumulation of EGCG and ECG under elevated CO_2_ conditions in tea leaves. As epicatechins constitute about 90% of total catechins in tea leaves, an enhancement in epicatechins content ultimately increased total catechins content under elevated CO_2_^28^. In albino tea plants, the expression levels of *CsPAL*, *CsF3H* and *CsFLS* are correlated with the endogenous concentration of catechins, where PAL is considered as a core regulator that controls biosynthesis of catechins^29^. In our study, elevated CO_2_ which is an important environmental cue, might directly or indirectly influence the transcription of all key genes of catechins biosynthetic pathway including *CsPAL* and thus resulted in increased levels of epicatechins and total catechins in tea leaves (Fig. 4C).

Furthermore, total amino acid and theanine concentrations increased in tea leaves when grown under elevated CO_2_ condition (Fig. 4, Table 2). The concentration of theanine is closely associated with the expression of its key biosynthetic genes namely *TS1* and *TS2* that encode theanine synthetase^30^. In addition, other two enzymes such as glutamine synthetase (GS) and glutamine: 2-oxoglutarate aminotransferase (GOGAT) catalyze the initial steps of NH_3_ assimilation into glutamic acid, are also considered as key determinant of theanine biosynthesis. In the current study, elevated CO_2_ sharply induced gene expression levels of *TS* and *GS* in tea leaves, which eventually resulted in increased theanine concentration as compared with that in ambient CO_2_-grown tea plants. Environmental stresses such as salt treatment could influence theanine biosynthesis. Increased theanine content under salt treatment was found to be associated with increased expression of theanine synthetase protein in tea leaves^31^. qPCR data of theanine biosynthetic genes are well in accord with the content of theanine (Fig. 6, Table 2). For multi-faceted health benefits of theanine, high concentration of theanine in tea leaves is considered as a sign of good quality. Our results suggest that CO_2_ enrichment can be considered as a potential approach to enhance theanine concentration in tea.

By way of contrast, the caffeine content was dramatically decreased following exposure of tea plants to elevated CO_2_. Caffeine is N-rich secondary metabolite, and its biosynthesis depends on the flow of N-based compounds toward secondary metabolic pathway^12^. Previous studies showed that elevated CO_2_ sharply decreased the levels of N-rich secondary metabolites such as nicotine at limited N-supply in tobacco^10^. This effect was presumably related to changes in primary nitrogen metabolism, as elevated CO_2_ typically decreased nitrate, ammonium, amino acids and protein under low and intermediate N-supply. Although, we noticed a sharp decrease in concentration of caffeine and total nitrogen at elevated CO_2_ grown tea plants, concentration of total amino acids increased in tea leaves (Figs 3D and 4A and D). The possibility of direct or indirect suppression of caffeine synthesis due to altered N metabolism under elevated CO_2_ cannot be ignored. Previous reports also showed that shading substantially increased caffeine content in tea leaves, implying that environmental cue has remarkable effect on the biosynthesis of caffeine^11^. From qPCR analysis, it becomes evident that elevated CO_2_ sharply down-regulated key genes involved in the biosynthesis of caffeine. Suppression of *sAMS* could suppress methylation steps of caffeine biosynthesis. Because SAM functions as methyl donor in the three methylation steps (Xanthosine to 7-methylxanthosine, 7-Methylxanthine to theobromine and finally theobromine to caffeine) in the caffeine biosynthetic pathway (Fig. 7), whereas SAM is converted to S-adenosyl-L-homocysteine (SAH)^12^. Similarly, down-regulation of *TIDH* and *TCS1*, the first and the last regulatory genes in caffeine biosynthesis under elevated CO_2_ further confirmed the potential reasons of decreased caffeine concentration under elevated CO_2_ condition in tea leaves.

To sum up, elevated CO_2_ induced photosynthesis and subsequently contents of carbohydrates such as starch, sucrose and sugar (Figs 1 and 3). At the same time, respiration was also induced by elevated CO_2_ (Fig. 2). Since carbohydrate is utilized in the process of respiration to produce energy, pyruvate and some other intermediates^8^, which are used in some anabolic pathways such as biosynthesis of amino acid, it is highly possible that an increase in respiration eventually stimulates amino acid biosynthesis. Here, the contents of EGC, EGCG and theanine were induced by elevated CO_2_, while content of caffeine was decreased (Fig. 4). It is interpreted in the carbon-nutrient balance hypothesis that under elevated CO_2_, excess carbon products that are not required for primary metabolic functions, will be allocated for biosynthesis of secondary metabolites, which eventually result in increased carbon-based secondary metabolites and subsequently decreased N-based secondary metabolites in plants^32^. These also explain a potential reason of elevated CO_2_-induced increased catechins and decreased caffeine concentrations in our current study. Results of qPCR analysis of catechins, theanine and caffeine biosynthetic genes were in good agreement with the biochemical data (Figs 5–7). As low caffeine and high theanine contents are desired for a better quality tea, it is quite possible that rising CO_2_ may improve green tea quality in the face of climate change. It will be interesting to further explore the molecular mechanisms that cause such biochemical changes in tea leaves under elevated CO_2_ condition.

## Materials and Methods

### Plant material and growth conditions

Seedlings of Longjing 43, a well-known green tea (*Camellia sisnensis* L.) cultivar, were grown in pots. Two years old tea seedlings were exposed to atmospheric CO_2_ at either 380 μmol mol^−1^ or 800 μmol mol^−1^, corresponding to the “ambient CO_2_” and “elevated CO_2_” treatments, respectively, in controlled-environment growth chambers (Conviron, Winnipeg, Canada). The growth conditions were as follows: the photosynthetic photo flux density (PPFD)- 600 μmol m^−2^ s^−1^, photoperiod- 14/10 h (day/night), day/night air temperature- 26/22 °C and relative humidity- 80%. CO_2_ enrichment treatment lasted for 24 day, while data for photosynthesis- and respiration-related parameters were recorded at 0, 6, 12, 18 and 24 day. There were 80 seedlings under each treatment, which were placed in four randomized blocks, representing four replicates. Thus, each replicate consisted of 20 pots. Pot placement within specified CO_2_ condition was randomized every 2 day. Meanwhile, seedlings were fertilized with Hoagland’s nutrient solution every 2 day. For harvesting samples, young leaves were collected from each block and pooled together separately.

### Estimation of photosynthesis, RuBisCO carboxylation capacity and Φ_PSII_

Net CO_2_ assimilation rate (Pn) was measured on 3^rd^ fully expanded leaves using an open-flow infrared gas analyzer adapted with light and temperature control systems (Li-COR 6400, Li-COR, Lincoln, NE, USA). Following method of von Caemmerer and Farquhar^33^, rate of CO_2_ assimilation/intercellular CO_2_ concentration (*A/Ci*) curves were measured in which the leaf temperature and PPFD were maintained at 25 °C and 1800 µmol m^−2^ s^−1^, respectively. The maximum carboxylation rate of RuBisCO (*V*_*cmax*_) and maximum rates of RuBP regeneration (*J*_*max*_) were estimated by fitting a maximum-likelihood regression below and above the inflexion of the *A/Ci* response according to the method described by Ethier and Livingston^34^.

The photochemical efficiency of photosystem II (Φ_PSII_) was determined by an imaging pulse amplitude modulated (PAM) fluorimeter (IMAG-MAXI; Heinz Walz, Effeltrich, Germany) and calculated according to Genty *et al*.^35^.

### Measurement of leaf respiration by O_2_ uptake

To determine leaf respiration, the O_2_ uptake by leaf segments was measured using a Clark-type liquid-phase oxygen electrode (Oxygraph-lab, Hansatech, UK)^36^. In brief, the plants were dark adapted for 30 min to avoid any light-enhanced photosynthesis; afterward, 0.1 g leaf samples were cut into pieces for measuring respiration at 25 °C in 2 mL of air-saturated 20 mM potassium phosphate buffer (pH 6.8). When oxygen uptake reached a constant rate, potassium cyanide (1 mM) or salicylhydroxamic acid (SHAM, 20 mM) was added for the estimation of cyanide (CN)- or SHAM-resistant respiration, respectively. Likewise, when a constant rate of O_2_ uptake was attained in the buffer without any reagents, the sucrose-induced leaf respiration was analyzed by adding 110 mM sucrose^37^.

### Determination of tea polyphenols and total amino acids quantification

The harvested leaf samples were immediately placed into an oven run at 105 °C for 15 min and then transferred to 80 °C until they were completely dried. The powdered dry samples were used for determination of tea polyphenols and amino acids. Total tea polyphenols was extracted and determined spectrophotometrically according to the method described by the International Organization for Standardization (ISO) 14502-1^38^. Gallic acid was used as standard. Briefly, the diluted sample extract (1.0 mL) was transferred to tubes in duplicate, where each tube contained 5.0 mL of a 1/10 dilution of Folin-Ciocalteu’s reagent in water. Afterward, 4.0 mL sodium carbonate solution (7.5% w/v) was added into each tube. The tubes were kept at room temperature for 60 min before absorbance at 765 nm was measured against water.

Amino acids from tea leaf sample (0.5 g) were extracted in 80% ethanol at 80 °C. Following evaporation, dried samples were dissolved in 0.02 N HCl. Amino acid content was determined using a Hitachi L-8900 amino acid analyzer (Hitachi, Japan). In brief, amino acids, separated by cation-exchange chromatography, were subjected to postcolumn reaction with ninhydrin reagent and detected spectrophotometrically as described previously elsewhere^39^.

### Quantification of catechins, caffeine and individual amino acids

The concentrations of caffeine and catechins in the extract was determined with a HPLC system (Waters 590, Waters Corp., Milford, MA, USA) equipped with a Hypersil ODS2 C18 column (5 ml, 4.6 mm × 250 mm, 35 °C) at 280 nm as previously described^39^. Solvents A (2% acetic acid) and B (acetonitrile) were run in linear gradients with A decreasing from 93% to 55% within 20 min and maintained for 5 min thereafter at a rate of 1.4 mL min^−1^. The concentrations of caffeine and catechins were quantified by their peak areas against those of standards prepared from authentic compounds.

An automatic amino acid analyzer (Hitachi L-8900, Japan) was used to measure individual amino acids including theanine (Thea), phenylalanine (Phe), aspartic acid (Asp), arginine (Arg), threonine (Thr), serine (Ser), valine (Val), alanine (Ala), proline (Pro) and γ-aminobutyric acid (GABA). Amino acids were measured by adding 5 mL of tea extract with 5 mL of sulfosalicylic acid and centrifuging the mixture at 13000 rpm for 5 min to facilitate the reaction. The mixture was filtered through a 0.20 μm nylon filter membrane and run using the amino acid analyzer^40, 41^.

### Determination of sugar, starch, total C and total N concentration

Soluble sugar and starch concentrations were determined by anthrone colorimetry in a spectrophotometer (SHIMADZU UV-2550, Kyoto, Japan) as described by Buysse and Merckx^42^. Total C and total N were measured by Vario MAX CN analyzer (Elementar Co. Ltd., Germany).

### RNA isolation and real-time qPCR assay

Total RNA from tea leaves was isolated using TRIzol reagent (Invitrogen, Carlsbad, CA, USA) according to the manufacturer’s instruction. Genomic DNA in RNA samples was removed using a purifying column. Reverse transcription was done using Superscript II (Invitrogen) following the manufacturer’s protocol. The primers used for transcript analysis have been listed in Supplementary Table S1. qPCR analysis was carried out using the StepOnePlus Real-Time PCR system (Applied Biosystems, Foster City, CA, USA) with Power SYBR Green PCR Master Mix (Applied Biosystems). The PCR conditions consisted of denaturation at 95 °C for 3 min, followed by 40 cycles of denaturation at 95 °C for 30 s, annealing at 58 °C for 30 s and extension at 72 °C for 30 s. Transcript abundance was normalized to *actin*, and relative gene expression was calculated following formulae of Livak and Schmittgen^43^. Four biological replicates were used for qPCR analysis.

### Statistical analysis

At least four independent replicates were conducted for each determination. The data were subjected to analysis of variance using SAS 8.0 software package (SAS Institute, Cary, NC), and the means were compared using Tukey’s test at the *P* < 0.05 level.

## Electronic supplementary material

Supplementary information
