# Supplementary material for: Stimulation in primary and secondary metabolism by elevated carbon dioxide alters green tea quality in Camellia sinensis L
Source: Sci Rep. 2017 Aug 11;7:7937. doi: 10.1038/s41598-017-08465-1 (PMC5554289; doi:10.1038/s41598-017-08465-1)
Supplement: Supplementary file 1 — Supplementary information [file 41598_2017_8465_MOESM1_ESM.pdf]

Stimulation in primary and secondary metabolism by elevated carbon dioxide alters green tea quality in *Camellia sinensis* L.

Xin Li[1], Golam Jalal Ahammed[1, 2], Zhi-Xin Li[1,3], Lan Zhang[1], Ji-Peng Wei[1], Chen Shen[1,3], Peng Yan[1], Li-Ping Zhang[1], Wen-Yan Han[1,*]

[1] *Key Laboratory of Tea Biology and Resources Utilization, Ministry of Agriculture, Tea Research Institute, Chinese Academy of Agricultural Sciences, 9 Meiling Road, Hangzhou, 310008, P.R. China*

[2]*Department of Horticulture, Zijingang Campus, Zhejiang University, Yuhangtang Road 866, Hangzhou, 310058, P.R. China*

[3]*Graduate School of Chinese Academy of Agricultural Sciences, Beijing, P. R. China*

* Corresponding author: **Wen-Yan Han**

E-mail address: hanwy@tricaas.com (W.-Y. Han)

Supplemental Table S1. Primers used for real time qPCR assays and treatment response (%) of transcripts whose abundance was significantly altered in tea seedlings grown at elevated CO_2 concentration (800 µmol mol^{-1}) compared to ambient CO_2 concentration (380 µmol mol^{-1}).

**Gene**	**Functional annotation**		**Primer pairs**	**% (x 100)**	***p***
Actin		U60481.1	F: 5’-CTCAGCACATTCCAGCAGAT-3’<br>R: 5’- ACTGCAGCTTTCCCAGAAAT-3’		
CsPAL	*PHENYLALANINE AMMONIA-LYASE*	D26596	F: 5’-GAATGCCGGTCTTATCCACT-3’<br>R: 5’-CGGTGAACACCTTGTCAAAC-3’	5.14	0.137
CsC4H	*CINNAMATE 4-HYDROXYLASE*	AY641731	F: 5’-CGAGAGGTTCTTGGAAGAGG-3’<br>R: 5’-AGAATTGGCAGAGCAAGGAT-3’	3.37	0.119
Cs4CL	*P-COUMARATE:COA LIGASE*	DQ194356	F: 5’-GGAGGTTATCCTGGACCTCA-3’<br>R: 5’-GGCAAGCCTTGTAGTGTGAA-3’	3.09	0.216
CsCHS	*CHALCONE SYNTHASE*	AY169403	F: 5’-GGCAATCAAAGAATGGGG-3’<br>R: 5’-ATGGGCGAAGACCGAGT-3’	2.44	0.102
CsCHI	*CHALCONE ISOMERASE*	DQ904329	F: 5’-CACAAAGAAGATTATGGGTGAAG-3’	6.19	0.331

			R: 5’-CAAACCTAGAAGTTGCCAAGAGT-3’		
CsF3H	*FLAVANONE 3-HYDROXYLASE*	AY641730	F: 5’-CTACTCAAGATGGCCCGACAA-3’<br>R: 5’-ACAACACCTCCAGCAACTTGC-3’	1.85	0.102
CsDFR	*DIHYDROFLAVONOL 4-REDUCTASE*	AB018685	F: 5’-ATTGGCAGAGAAAGCAGCAT-3’<br>R: 5’-GTGATTAGGCTTGGTGGGAA-3’	3.29	0.219
CsANS	*ANTHOCYANIDIN SYNTHASE*	AY830416	F: 5’-GGCCACAAGTGCCTACAATTG-3’<br>R: 5’-CCCATGATTCACCAAATGCA-3’	2.38	0.122
CsUFGT	*UDP- GLUCOSE FLAVONOID 3-O-GLUCOSYL TRANSFERASE*	GH618818	F: 5’-GGCAAGAAGCTAATAGGGTCGTT-3’<br>R: 5’-TTGTATCATTCGGAAGTGgTGGG-3’	3.46	0.203
CsANR	*ANTHOCYANIDIN REDUCTASE*	AY641729	F: 5’-GCGAAGTTGATCCTCTCGTC-3’<br>R: 5’-AACCACATCGTCAAGTGAACA-3’	5.35	0.190
CsLAR	*LEUACOANTHOCYANIDIN REDUCTASE*	AY169404	F: 5’-GAATGCCGGTCTTATCCACT-3’<br>R: 5’-CGGTGAACACCTTGTCAAAC-3’	0.622	0.016
CsTIDH	*INOSINE 5’-MONOPHOSPHATE DEHYDROGENASE*	EU106658	F: 5’-TGCTGAAAGAAGTGGTGTGC’<br>R: 5’-TCCCTTTTGTCATTGCTTCC-3’	0.195	0.006
CssAMS	*S-ADENOSYL-L-METHIONINE SYNTHASE* (*sAMS*)	AB041534	F: 5’-ACCCAACACGATGAGACCG-3’<br>R: 5’-GCCAATGACAAAGCGACCA-3’	0.634	0.027
CsTCS1	*TEA CAFFEINE SYNTHASE 1*	AB031280	F: 5’-CTGTCGTCTGAGGTTATTGG-3’	0.389	0.011

			R: 5'-GTGCCTGAGTAAGCCAATGA-3'		
CsGS1	*GLUTAMINE SYNTHETASE*	AB115184	F: 5'-CCCTCTGCTTACGCTCTGT-3'<br>R: 5'-GCTTTTTGGGATCATTAACG-3'	2.13	0.091
CsGS2	*GLUTAMINE SYNTHETASE*	AB117934	F: 5'-GTTTGTTTGGAGTGGGTGT-3'<br>R: 5'-AGTGCTGGAACCATCGTAG-3'	0.472	0.017
CsGS	*GLUTAMINE SYNTHETASE*	EU284131	F: 5'- GGAGGTTATCCTGGACCTCA-3'<br>R: 5'- GGCAAGCCTTGTAGTGTGAA-3'	3.48	0.274
CsGOGAT	*GLUTAMINE: 2-OXOGLUTARATE AMINOTRANSFERASE*	JN602371	F: 5'-ACACTGCCACATCTCAAAGG-3'<br>R: 5'-CCAATTGATCAGCATTGACC-3'	0.632	0.039
CsTS1	*THEANINE SYNTHASE*	DD401895	F: 5'-TCTTTCTGGACCTGTGAGTG-3'<br>R: 5'-GCTTGAGGGTAGATAATGAGT-3'	3.70	0.288
CsTS2	*THEANINE SYNTHASE*	DD401896	F: 5'-AAGGACCCATTCAGGAGAGG-3'<br>R: 5'-CAATCGGCCACTTCACTTCT-3'	0.464	0.035
